# Supplementary figures and images for: Efficacy of tenapanor in managing hyperphosphatemia and constipation in hemodialysis patients: A randomized controlled trial
Source: PLoS One. 2025 Jun 17;20(6):e0319319. doi: 10.1371/journal.pone.0319319 (PMC12173349; doi:10.1371/journal.pone.0319319)

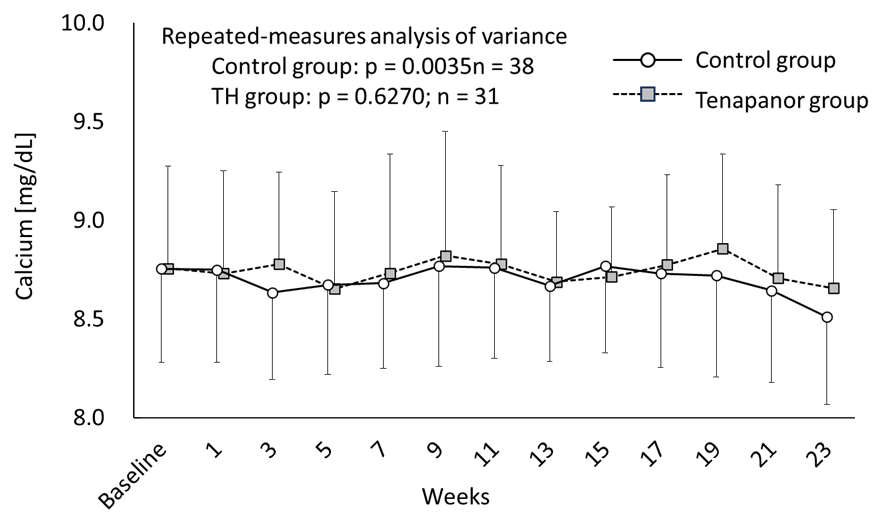

Supplement: S1 Fig — The data are shown as the mean ± SD. (TIF) [file pone.0319319.s001.tif]

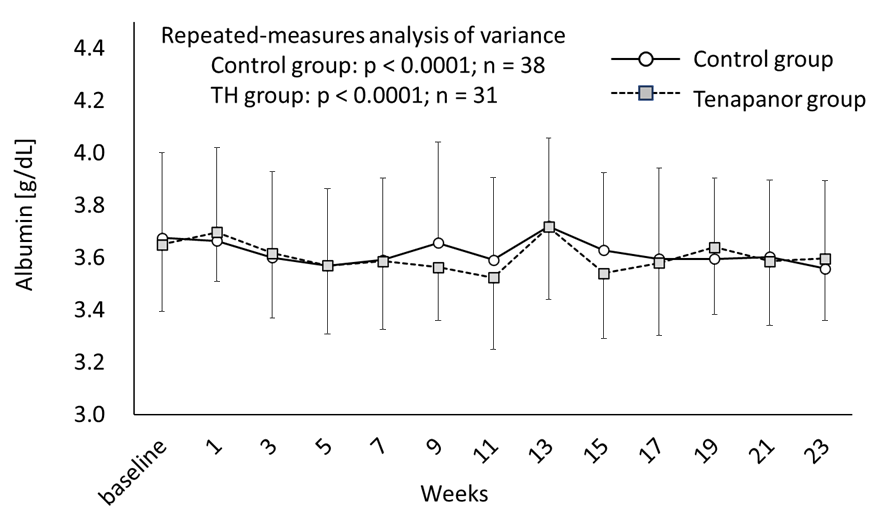

Supplement: S2 Fig — The data are shown as the mean ± SD. (TIF) [file pone.0319319.s002.tif]
