# Supplementary material for: Efficacy of tenapanor in managing hyperphosphatemia and constipation in hemodialysis patients: A randomized controlled trial
Source: PLoS One. 2025 Jun 17;20(6):e0319319. doi: 10.1371/journal.pone.0319319 (PMC12173349; doi:10.1371/journal.pone.0319319)
Supplement: S1 Table — (DOCX) [file pone.0319319.s003.docx]

| Variables | Overall (n = 69) | Tenapanor (n = 31) | Control (n = 38) | p value |
| --- | --- | --- | --- | --- |
| Age, years | 72.0±9.4 | 73.9±9.2 | 70.4±9.3 | 0.122 |
| Male, n (%) | 35(50.7) | 16(51.6) | 19(50.0) | 1.000 |
| Hemodialysis vintage, years | 14.0(2.0-22.0) | 17.0(2.5-24.0) | 12.0(2.3-21.0) | 0.781 |
| Diabetes mellitus, n (%) | 24(34.8) | 12(38.7) | 12(31.6) | 0.615 |
| Dry weight, kg | 55.7±11.9 | 55.6±11.6 | 55.8±12.3 | 0.955 |
| Hemoglobin, g/dL | 11.2±1.0 | 11.2±1.2 | 11.2±0.9 | 0.949 |
| Urea nitrogen, mg/dL | 62.9±13.9 | 62.8±14.0 | 63.0±14.1 | 0.975 |
| Creatinine, mg/dL | 9.72±2.09 | 9.86±1.90 | 9.60±2.25 | 0.612 |
| Calcium, mg/dL | 8.76±0.50 | 8.76±0.53 | 8.76±0.48 | 0.982 |
| Inorganic phosphorus, mg/dL | 5.11±1.18 | 5.17±1.37 | 5.07±1.02 | 0.707 |
| Albumin, g/dL | 3.67±0.30 | 3.65±0.26 | 3.68±0.33 | 0.706 |
| C-reactive protein, mg/dL | 0.08(0.05-0.15) | 0.09(0.05-0.15) | 0.08(0.05-0.15) | 0.885 |
| Intact parathyroid hormone, pg/mL | 102.0(55.0-183.0) | 79.0(48.0-218.0) | 108.0(81.3-174.5) | 0.866 |
| Single pool Kt/V urea | 1.83±0.36 | 1.76±0.34 | 1.76±0.34 | 0.128 |

**S1 Table.** Baseline characteristics of participants who completed the study (n = 69).
