# Supplementary material for: Efficacy of tenapanor in managing hyperphosphatemia and constipation in hemodialysis patients: A randomized controlled trial
Source: PLoS One. 2025 Jun 17;20(6):e0319319. doi: 10.1371/journal.pone.0319319 (PMC12173349; doi:10.1371/journal.pone.0319319)
Supplement: S2 File — (PDF) [file pone.0319319.s005.pdf]

The effect of tenapanol hydrochloride on serum phosphorus levels and stool Consistency

- ## Purpose of the study

Tenapanor (Fosebel®, Kyowa Hakko Kirin), which is expected to be launched as a new phosphate binder in February 2024, is a drug that inhibits Na+/H+ exchanger 3 (NHE3) and has a different mechanism of action than conventional phosphate binders. Tenapanor reduces Na+ absorption and increases H+ absorption in the gastrointestinal tract. This lowers the pH within the intestinal epithelial cells and inhibits intestinal absorption of phosphate. It was initially noted as a treatment for constipation-type irritable bowel syndrome, but it has also been shown to inhibit passive phosphate absorption in the intestine and is also considered promising for the treatment of hyperphosphatemia.

Currently used phosphate binders include calcium-based binders, such as calcium carbonate, and non-calcium-based binders, such as sevelamer and lanthanum carbonate. However, these phosphate binders are likely to cause gastrointestinal symptoms, and the increased burden of taking the medication may lead to a decrease in patient adherence. In addition, there are concerns about the long-term safety of lanthanum carbonate, which has been reported to form lanthanum deposits in the stomach and duodenum.

In previous studies, diarrhea was the most commonly reported gastrointestinal adverse event with tenapanor, but the majority of cases were mild and the incidence rate was reported to be between 63.7% and 74.4%. On the other hand, it is believed that the effect of tenapanor on the intestinal environment may improve constipation in dialysis patients and reduce the amount of laxatives used. We hypothesized that long-term use of Tenaponar would normalize stool consistensy in dialysis patients and reduce laxative use. In this study, we will conduct a randomized controlled trial to evaluate the effect of Tenaponar on serum phosphorus levels and stool consistency in a real-world clinical setting to test this hypothesis.

- ## Principal Researcher

Affiliation: Department of Clinical Engineering
Name: Naoki Suzuki

- **Study design**: open-label randomized controlled trial

- ## Randomization method

Patients will be stratified by baseline serum phosphate level (≤5.5 mg/dL or >5.5 mg/dL) and randomized 1:1 to tenapanor or standard of care using a computer-generated sequence.

- **Observation period**: 24 weeks (6 months) from the start of Tenapanor hydrochloride (TH) administration

- **Place of implementation:** Dialysis room at Tojinikai Hospital Annex Clinic

- **Target number of subjects:** Tenapanor group: 50, control group: 50

- **Exclusion criteria**
  - Patients who have been on dialysis for less than 2 years
  - Patients with a history of inflammatory bowel disease or diarrhea-type irritable bowel syndrome
  - Patients with a C-reactive protein level of 1.0 mg/dL or higher

- **Teanapanor dosage**

  The dosage should be started at 10 mg/day and adjusted according to the serum phosphorus level. The dosage should be determined by the attending physician.

- **Primary outcome**

  Changes in serum phosphorus levels and effects on stool consistency due to Tenapanore administration.

- **Secondary outcome**

  Adverse events related to tenapanor use, changes in number of laxative prescriptions.

- **Evaluation Items**

- Main outcome measures
  - ✓ BSFS from baseline to 7 weeks after dosing (every week)
  - ✓ Serum phosphorus levels from baseline to 23 weeks after dosing (every 2 weeks with regular blood sampling)*.

- Secondary outcome measures
  - ✓ Patient background at baseline
  - ✓ Tenapanor dosage from baseline to week 23 (every week until week 11, every 2 weeks starting at week 11)
  - ✓ Types of laxatives prescribed from baseline through week 7 and week 23 after treatment
  - ✓ Serum calcium and albumin levels from baseline through week 23 of treatment (regular blood draws every 2 weeks)*.
  - ✓ Number of bowel movements from baseline through week 7 of treatment (every week)
  - ✓ Adverse events (AEs) from baseline through 7 weeks after dosing (nausea,

vomiting, diarrhea, abdominal pain, fatigue, other) (every week)

*, Comparison between tenapanor group and control group

- Statistical analysis
  - Baseline characteristics will be compared using Welch's t-test for continuous variables and chi-square test for categorical variables.
  - Changes in blood data in the tenapanor and control groups will be analyzed using repeated measures analysis of variance.
  - Changes in variables in the tenapanor group (proportion of phosphate binders prescribed, BSFS score, and proportion of laxatives prescribed) will be visualized using a Sankey diagram. Values that follow a normal distribution will be reported as mean ± standard deviation, and values that do not follow a normal distribution will be presented as median and interquartile range.
  - The magnitude of the difference in effect size will be evaluated using Cohen's d.
  - A p-value of less than 0.05 will be considered statistically significant.
  - The analysis will be conducted using R software (version 4.0.3).

- Ethical considerations in medical research and practice directly involving the human body
  - Protecting the human rights of human subjects of medical research and practice

    The researchers in this study will conduct this research in accordance with the Declaration of Helsinki. The information of the research subjects will be used in the study after the researchers in charge of the study have anonymized it so that individuals cannot be identified from the medical records. The anonymization table and data will be stored on a PC with access rights managed by the principal investigator. Paper records will be stored in a locked cabinet.

  - Advantages and Disadvantages for Individuals Subject to Medical Research and Treatment

    This research will be conducted in the context of medical treatment and there are no anticipated adverse events. There are also no benefits, such as compensation, for the research subjects.

  - Medical Contribution

    Tenapanor is a new drug that will be launched in February 2024, and there are few studies of it in actual clinical practice in Japan other than clinical trials. As a result, there are few reports on its efficacy compared with other drugs, and the

background factors of cases in which it is effective and cases in which it is not effective have not been clarified. In addition, the changes in stool consistency and the amount of laxative prescribed for tenapanor in dialysis patients have not been clarified. Therefore, this study is highly novel, and it is believed that it will provide results that can be widely applied to daily medical practice.

- Method of obtaining the understanding and consent of persons who are subjects of medical research and medical treatment

  The subjects will be informed in accordance with the explanatory document prepared this time by the test collaborators, and it will be a condition for the introduction of this test that consent will be obtained by means of a consent form after the subjects have fully understood 1) the content and necessity of the survey, 2) that there will be no disadvantage due to the presence or absence of consent, 3) that it is possible to withdraw at any time even after consent has been given, and 4) the measures to ensure the confidentiality and anonymity of the data. In principle, the consent of the individual is required, but if consent cannot be confirmed for any reason, the test will be introduced with the consent of a guardian, etc.

Evaluation Schedule Table

Evaluation Items	Weeks																										
	-3	-2	-1	0	1	2	3	4	5	6	7	8	9	10	11	12	13	14	15	16	17	18	19	20	21	22	23
	0	1	2	3	4	5	6	7	8	9	10	11	12	13	14	15	16	17	18	19	20	21	22	23	24	25	26
	2/19	2/25	3/4	3/11	3/18	3/25	4/1	4/8	4/15	4/23	4/29	5/6	5/13	5/20	5/27	6/3	6/10	6/17	6/24	7/1	7/8	7/15	7/22	7/29	8/5	8/12	8/19
Dosage					○	○	○	○	○	○	○	○	○	○	○		○		○		○		○		○		○
Blood data				○	○		○		○		○		○		○		○		○		○		○		○		○
BSFS				○	○	○	○	○	○	○	○																
Number of bowel movements	Preparation period			○	○	○	○	○	○	○	○																
Laxative				○							○																○
Adverse event				○	○	○	○	○	○	○	○																

* Tenapanore dosage: 0-11 weeks, every 1 week; 11 weeks and after, every 2 weeks
* Blood data: 0-23 weeks, every 2 weeks (regular blood sampling)
* Laxatives: 0, 7 and 23 weeks
* BSFS, number of bowel movements, adverse events: 0-7 weeks, every 1 week
