# Supplementary material for: Efficacy of tenapanor in managing hyperphosphatemia and constipation in hemodialysis patients: A randomized controlled trial
Source: PLoS One. 2025 Jun 17;20(6):e0319319. doi: 10.1371/journal.pone.0319319 (PMC12173349; doi:10.1371/journal.pone.0319319)
Supplement: S3 File — (PDF) [file pone.0319319.s006.pdf]

2024/1/20

テナパノル塩酸塩の血清リン値と便性状への影響

- 研究の目的

　2024 年 2 月に新たなリン吸着薬として発売される予定のテナポナール（フォゼベル®、協和発酵キリン）は、Na+/H+交換輸送体 3（NHE3）を阻害する薬剤であり、従来のリン吸着薬とは異なる作用機序を有する。テナポナールは、消化管での Na+吸収を低下させ、H+吸収を増加させる。これにより腸管上皮細胞内の pH が低下し、リン酸の腸管吸収が抑制される。当初は便秘型過敏性腸症候群の治療薬として注目されていたが、腸管での受動的リン酸吸収を抑制する能力が示され、高リン酸血症治療にも有望とされている。

　現在使用されているリン結合剤には、炭酸カルシウムなどのカルシウム系と、セベラマーや炭酸ランタンなどの非カルシウム系がある。しかし、これらのリン結合剤は消化器症状を引き起こしやすく、服薬負担の増大により患者の服薬アドヒアランスが低下する可能性がある。また、炭酸ランタンには長期安全性に懸念があり、胃や十二指腸にランタン沈着物を形成することが報告されている。

　これまでの研究では、テナポナールの胃腸における有害事象として下痢が最も多く報告されているが、大半は軽度であり、発生率は 63.7%～74.4%とされている。一方で、テナポナールが腸管環境に与える影響により、透析患者の便秘が改善し、下剤使用量が減少する可能性があると考えられる。我々は、テナポナールの長期使用が透析患者の便性状を正常化させ、下剤使用量を減少させるという仮説を立てた。本研究では、この仮説を検証することを目的として、実際の臨床現場におけるテナポナールの血清リン濃度および便性状への影響を評価する無作為化比較試験を実施する。

- **研究責任者**：

所属：臨床工学部
氏名：鈴木　尚紀

- **研究デザイン**：非盲検化無作為化比較試験

- **無作為化の方法**

　ベースラインの血清リン酸レベル（≦5.5 mg/dL または>5.5 mg/dL）で層別化し、コンピュータ生成のシーケンスを用いて、テナポナールまたは標準治療のいずれかに 1:1 の

割合で割り付ける。

- **観察期間**：テナパノル塩酸塩（TH）投与開始より 24 週（6 カ月）
- **実施場所**：桃仁会病院付属診療所透析室
- **目標対象数**：tenapanor 群：50 名、Control 群：50 名
- **除外基準**
  - ➢ 透析導入後 2 年未満の患者
  - ➢ 炎症性腸疾患又は下痢型過敏性腸症候群の合併又は既往歴のある患者
  - ➢ C 反応性蛋白（CRP）が 1.0mg/dL 以上の患者
- **Teanapanor 投与量**

  一日 10 mg/day から投与を開始し、血清リン値によって投与量を増減する。投与量は各主治医が決定する。
- **主要評価項目**

  TH 使用による血清リン値の推移と便性状への影響
- **副次評価項目**

  tenapanor 使用による有害事象、便秘薬処方数の変化
- **評価項目**

➢ 主要評価項目
  - ✓ ベースラインから投与後 7 週後までの BSFS（1 週毎）
  - ✓ ベースラインから投与後 23 週後までの血清リン値（2 週毎定期採血）※

➢ 副次評価項目
  - ✓ ベースラインの患者背景※

    ベースラインから 23 週までの tenapanor 投与量（11 週までは 1 週毎、11 週以降は 2 週毎）
  - ✓ ベースラインから投与後 7 週後、23 週後の便秘薬処方種類
  - ✓ ベースラインから投与後 23 週後までの血清カルシウム、アルブミン濃度（2 週毎定

期採血）※

- ✓ ベースラインから投与後 7 週後までの排便回数（1 週毎）
- ✓ ベースラインから投与後 7 週後までの有害事象（悪心・嘔吐・下痢・膨満感・胃痛・倦怠感・その他）（1 週毎）

※は Tenapanor 群とコントロール群で比較

● **統計解析**

- ➢ ベースラインの特性は、連続変数については Welch の t 検定を、カテゴリー変数についてはカイ二乗検定を用いて比較する。
- ➢ Tenapanor 群と Control 群における血液データの変化は、反復測定分散分析を用いて分析する。
- ➢ Tenapanor 群における変数（リン結合剤処方の割合、BSFS スコア、および下剤処方の割合）の変化は、サンキーダイアグラムを用いて視覚化する。正規分布する値は平均値±標準偏差で報告し、正規分布しない値は中央値と四分位範囲で提示する。
- ➢ 効果量の差の大きさは Cohen’s d を用いて評価する。
- ➢ p 値が 0.05 未満の場合に統計的に有意と判断する。
- ➢ 解析は R ソフトウェア（バージョン 4.0.3）を用いて実施する。

● **人体を直接対象とした医学研究ならびに医療行為における倫理的配慮について**

➢ 医学研究及び医療行為の対象となる個人の人権の擁護

　本研究の研究者はヘルシンキ宣言に従って本研究を実施する。研究対象者の情報は、医療記録から個人が特定できないように、研究担当者が連結可能匿名化したうえで研究に使用する。匿名化の対応表及びデータは研究責任者がアクセス権の管理された PC に保存する。紙資料は鍵の掛かるキャビネットに保管する。

➢ 医学研究及び医療行為の対象となる個人への利益と不利益

　本研究は保険診療内での研究であり、想定される有害事象はないと考えられる。また、研究対象者への補償などの利益はない。

➢ 医学的貢献度

　Tenapanor は、2024 年 2 月に発売される新薬であり、国内において治験以外における実臨床での研究は少ない。その為、他の薬剤と比較した有効性の報告も少なく、効果が得られる症例及び得られない症例の背景因子も明らかにされていない。また、透

析患者における tenapanore の便性状への変化と便秘処方量についても明らかにされていない。従って、本検討は新規性が高く、日々の診療に広く貢献できる知見が得られると考えられる。

- 医学研究及び医療行為の対象となる個人に理解を求め同意を得る方法

　対象者には試験協力者が今回作成した説明書に沿って説明し，1) 調査の内容・必要性，2) 同意の有無による不利益は生じない，3) 同意後であっても随時撤回が可能である事、4) データの機密性と匿名性を確保するための措置についてについて、十分理解いただいたうえで，同意書を用いた同意取得を本試験導入の条件とする．本人の同意を原則とするが，何らかの理由により同意の意思が確認できない場合，後見人等の同意をもって試験導入とする。

評 価 予 定 表

評価項目	Weeks																										
	-3	-2	-1	0	1	2	3	4	5	6	7	8	9	10	11	12	13	14	15	16	17	18	19	20	21	22	23
	0	1	2	3	4	5	6	7	8	9	10	11	12	13	14	15	16	17	18	19	20	21	22	23	24	25	26
	2/19	2/25	3/4	3/11	3/18	3/25	4/1	4/8	4/15	4/23	4/29	5/6	5/13	5/20	5/27	6/3	6/10	6/17	6/24	7/1	7/8	7/15	7/22	7/29	8/5	8/12	8/19
TH 投与量					○	○	○	○	○	○	○	○	○	○	○		○		○		○		○		○		○
血液データ				○	○		○		○		○		○		○		○		○		○		○		○		○
BSFS		準備期間		○	○	○	○	○	○	○	○																
便回数				○	○	○	○	○	○	○	○																
便秘薬				○							○																○
有害事象				○	○	○	○	○	○	○	○																

■は tenapanore 投与前を示す。

※ TH 投与量：0-11w は 1w 毎、11w 以降は 2w 毎

※ 血液データ：0-23w まで 2w 毎（定期採血）

※ 便秘薬：0w、7w、23w

※ BSFS、便回数、有害事象：7w まで 1w 毎
